# Enzyme Replacement Therapy (ERT) on Heart Function Changes the Outcome in Patients with Infantile-Onset Pompe Disease: A Familial History

**DOI:** 10.1155/2023/8470341

**Published:** 2023-02-17

**Authors:** Marco Lecis, Katia Rossi, Maria Elena Guerzoni, Ilaria Mariotti, Lorenzo Iughetti

**Affiliations:** ^1^Post-Graduate School of Pediatrics, Department of Medical and Surgical Sciences for Mother Children and Adults, University of Modena and Reggio Emilia, Via Del Pozzo 71, Modena 41124, Italy; ^2^Neonatology Unit, Department of Medical and Surgical Sciences for Mother Children and Adults, University of Modena and Reggio Emilia, Via Del Pozzo 71, Modena 41124, Italy; ^3^Pediatrics Unit, Department of Medical and Surgical Sciences for Mother Children and Adults, University of Modena and Reggio Emilia, Via Del Pozzo 71, Modena 41124, Italy

## Abstract

**Background:**

Lysosomal acid alpha-glucosidase (GAA) deficiency, also known as Pompe disease, is an autosomal recessive disorder that leads to the accumulation of glycogen in lysosomes and cytoplasm, resulting in tissue destruction. Infantile-onset GAA deficiency is characterized by cardiomyopathy and severe generalized hypotonia. Without treatment, most patients die within the first two years of life. The demonstration of reduced GAA activity, followed by sequencing of the GAA gene, confirms the disease. GAA deficiency is currently treated with enzyme replacement therapy (ERT) with improved clinical outcomes and survival. *Case Presentation*. We describe the case of DGAA in two siblings, in which the diagnostic time point, treatment, and outcomes were completely different. The girl was diagnosed with DGAA at the age of 6 months during investigations for poor weight gain and excessive sleepiness. The finding of severe cardiomyopathy through EKG and echocardiography led to the suspicion of storage disease, and the GAA deficiency was later confirmed by genetic analysis. The girl died of complications due to the clinical picture before starting ERT. Conversely, her younger brother had the opportunity to receive an early diagnosis and the rapid onset of ERT. He is showing a regression of cardiac hypertrophy.

**Conclusion:**

The advent of ERT improved clinical outcomes and survival in infantile-onset PD. Its impact on cardiac function is still under study, but different reports in the literature have shown encouraging data. Early recognition of DGAA and prompt initiation of ERT is therefore crucial to prevent the progression of the disease and improve the outcomes.

## 1. Introduction

Lysosomal acid alpha-glucosidase (GAA, also called acid maltase) deficiency (Pompe disease, formerly classified as glycogen storage disease type II (GSD II)) is a rare autosomal recessive disorder with considerable allelic heterogeneity. It is caused by pathogenic variants in the gene for GAA. Deficiency of GAA (DGAA) leads to the accumulation of glycogen in lysosomes and cytoplasm, which results in tissue destruction, most affecting the skeletal muscle [1, 2]. Infantile-onset DGAA is characterized by cardiomyopathy and severe generalized hypotonia. Without treatment, most patients die within the first two years of life [3, 4]. Late-onset disease (juvenile and adult presentation) is characterized by skeletal myopathy (usually in a limb-girdle distribution) and a protracted course leading to respiratory failure without cardiomyopathy [5]. Infantile-onset DGAA should be suspected in infants with profound hypotonia and cardiac insufficiency [6, 7]. Juvenile or adult-onset DAA should be considered in patients with progressive weakness in a limb-girdle distribution [5]. Supportive findings may include an electrocardiogram (EKG) demonstrating a short PR interval and giant QRS complexes, suggesting biventricular hypertrophy, although this is a nonspecific finding (infantile-onset form) [6, 4]; electromyogram demonstrating myopathic discharge, sometimes associated with abundant myotonic and complex repetitive discharges, most prominent in the paraspinal muscles (late-onset form) [5]; elevated serum creatine kinase (CK; all forms) [5–7]. Demonstration of reduced GAA activity in dried blood spots or leukocytes, followed by sequencing of the GAA gene, confirms the disease [6]. DGAA deficiency is currently treated with enzyme replacement therapy (ERT), physical and occupational therapy, and supportive care (e.g., mechanical ventilation for respiratory failure) [8]. The advent of ERT improved clinical outcomes and survival for both early- and late-onset DGAA [9]. Here, we describe the case of two siblings whose fate was completely different and influenced by the possibility of making an early diagnosis and starting the ERT. We also offer a brief review of the literature on the cardiac involvement of PD and on the effects of ERT.

## 2. Case Reports

### 2.1. Case 1

**Figure 1:**
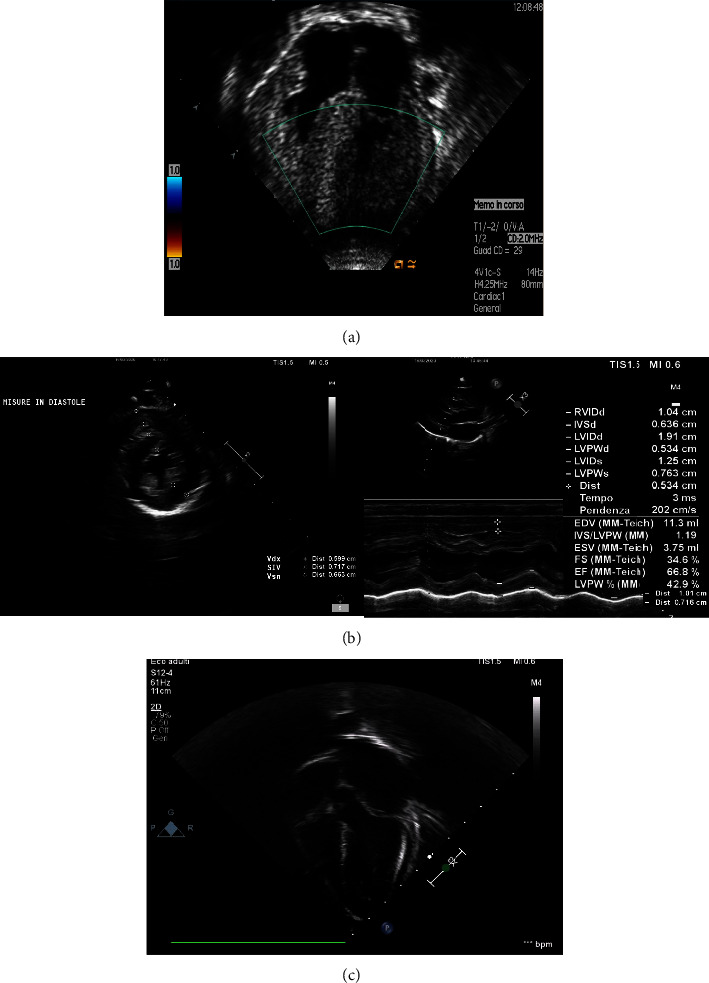
(a) Cardiac hypertrophy in a 6 months patient (XX) with DGAA; (b) cardiac hypertrophy in an infant (XY) with DGAA; (c) regression of cardiac hypertrophy in patient (XY) after 15 months of ERT.

A six-month-old female infant was referred to our attention for poor weight gain, poor food intake, and excessive sleepiness. Born at 40 weeks of gestational age, after a normal pregnancy, and characterized by maternal hypertension, his birth measurements were 2980 grams of weight, length 51 cm, head circumference 34 cm, and an Apgar score of 9-10. For detection of a perioral cyanotic mask occurring during and after the meal, echocardiography performed at 2 days of life showed normal parameters, apart from the size of the ventricles and septum at the upper limits of the norm. For this reason, a followup was scheduled at one month of life, which was not carried out due to noncompliance of the parents. At admission, her vital parameters were normal (SpO2 100%, HR 135 bpm, RR 47 bpm) and the clinical examination was notable for a slightly dystrophic appearance, small nose with anteverted nostrils, scaphocephaly, barrel chest, systolic murmur, axial hypotonus, and of the shoulder and pelvic girdles. The finding of severe left ventricular hypertrophy on EKG and a significant increase in CPK, GPT, and troponin I (CPK 557 U/L, CPK-MB 41.6 ng/ml, GPT 146 U/L, troponin I 0.31 ng/ml) raised the suspicion of congenital hypertrophic heart disease. A chest X-ray showed cardiomegaly and echocardiography confirmed the cardiomyopathy with the severe phenotypic expression of nonstructural hypertrophic type, with moderate reduction of the global contractile function of the left ventricle and acute heart failure (Figure 1(a)). With suspicion of storage disease, we performed genetic tests that confirmed the diagnosis of DGAA. Unfortunately, the child died shortly after from complications related to cardiac insufficiency.

### 2.2. Case 2

Five years later, the parents gave birth to a boy, born at 39 weeks from spontaneous birth, with a 10-10 Apgar score and normal anthropometric measures (3.450 gr of weight, 50 cm length, and 35 cm head circumference) without any dysmorphism. The neonatal course was characterized by hypoglycemia, hyponatremia, and hypocalcemia corrected with oral supplementation. In consideration of the family history of PD, a genetic analysis was performed which resulted positive for deletion of c.235-247 in homozygosity of exon 3 of the GAA gene. The infant was therefore diagnosed with PD and carried out the following investigations: blood tests (Aspartate Transaminase and CK increased, 53 IU/L and 1069 IU/L, respectively), EKG, echocardiography (myocardial hypertrophy of the left ventricle, with normal kinetics and contractility, Figure 1(b)), and alpha-glucosidase assay (0.25 *µ*mol/L/h, normal value > 1.45 *µ*mol/L/h). To initiate the enzyme replacement therapy, the evaluation of the CRIM (cross-reactive immunological material) status was also performed. Given the negative results, immunomodulatory therapy with Intravenous immunoglobulins (IVIGs) 500 mg/kg, Rituximab (12.5 mg/kg, 3 infusions), and methotrexate (0.4 mg/kg, 5 infusions) was initiated concurrently with the first infusions of alglucosidase alfa (Myozime, SANOFI) at starting dose of 20 mg/kg/week). The dosage of alglucosidase alfa was then increased to 40 mg/kg/week after 2 weeks and methotrexate was replaced with sirolimus (1 mg/m^2^/die) after three months. Periodic infusions of IVIG and blood tests were warranted, along with neuro-psycho-behavioral assessments, the latter showing a substantially adequate development for age. The child performed cadenced echocardiography, which showed a progressive improvement of the hypertrophic picture from the beginning of the ERT (Figure 1(c)). Currently, the child is 18 months old and is substantially in good health. He also passed without complications and a SARS-Cov2 infection occurred in October 2020 with febrile symptoms, but without clinically significant respiratory distress or cardiac sequelae.

## 3. Discussion

DGAA was the first identified lysosomal storage disease. Infants with infantile-onset DGAA typically have symptoms during the first few months of life. The classic infantile form is characterized by cardiomyopathy and severe, generalized muscular hypotonia. Clinical findings, signs, and symptoms include cardiomegaly (92%), respiratory distress (78%), muscle weakness (63%), feeding difficulties (57%), and failure to thrive (53%) [7]. Serum CK is typically elevated in DGAA and leukocyte GAA activity is usually decreased [6]. Moreover, elevations of CK, lactate dehydrogenase, and aspartate aminotransferase are commonly seen.

In infantile-onset DGAA, cardiac involvement is early and often severe and is one of the most important determinants of poor prognosis. It is characterized by hypertrophic cardiomyopathy. Thus, cardiac enlargement is found in thoracic radiography. EKG findings include short PR interval and high voltage QRS complexes in all leads because of left ventricular hypertrophy [10]. Furthermore, bradycardia in the newborn may disclose glycogenosis storage [11]. Echocardiography provides useful morphological information for the diagnosis of hypertrophic cardiomyopathy. Right and left ventricular walls are thickened [12]. The left ventricular cavity can be reduced because of severe wall hypertrophy leading to left ventricular outflow tract obstruction and abnormal ventricular compliance [13]. The diastolic function may be impaired because of left ventricular hypertrophy. In a Dutch patient group [14], the diastolic thickness of the left ventricular posterior wall and cardiac weight at autopsy were increased significantly with age.

The diagnosis of infantile-onset DGAA should be suspected in an infant with severe hypotonia and cardiac insufficiency. GAA enzyme activity can be measured in white blood cells or dried blood spots. Gene sequencing is the preferred test to confirm the diagnosis since it is routinely available, is less invasive, may provide genotype-phenotype information, and may help predict cross-reactive immunologic material (CRIM) status (amount of residual endogenous GAA production) in some cases [6].

The differential diagnosis for classic infantile disease with hypertrophic cardiomyopathy includes the following theories:

- Lysosome-associated membrane protein 2 deficiency, presenting with hypertrophic cardiomyopathy, muscle weakness, and hypotonia
- Fatty acid oxidation disorders, including very long-chainacyl-CoA dehydrogenase deficiency, long-chain 3-hydroxy-acyl-CoA dehydrogenase deficiency, carnitine transporter deficiency, carnitine-acylcarnitine translocase deficiency, and carnitine palmitoyltransferase deficiency type 2, presenting in infancy with hypertrophic cardiomyopathy with nonketotic hypoglycemia
- Mitochondrial and respiratory chain disorders, which may present with hypotonia, cardiomyopathy, hepatomegaly, and seizures
- Other infantile-onset hypotonia without cardiomyopathy, including spinal muscular atrophy type 1 and GSD type IIIa

Without treatment, most patients with the classic infantile form have unremitting deterioration with death during the first two years of age from cardiac insufficiency [7]. However, prolonged survival has been reported in infants with less severe cardiomyopathy [7].

The current treatment for DGAA is ERT with alglucosidase alfa. Standard dosing is 20 mg/kg given intravenously every two weeks. Dosing may be increased twofold to 20 mg/kg once a week or 40 mg/kg every two weeks in those with a poor response to initial therapy. A multidisciplinary care team is needed to obtain short- and long-term improvements in cardiac and skeletal muscle function and survival [9, 15–17].

New treatments such as gene therapy are under development to increase the intrinsic ability of the affected cells to produce GAA. Key components of gene therapy strategies include the choice of vector promoter and the route of administration. The efficacy of gene therapy depends on the ability of the vector to drive gene expression in the target tissue and on the recipient's immune tolerance to the transgene protein [18].

As noted above, cardiac involvement is one of the most important determinants of the poor prognosis of infantile-onset DGAA. However, different literature data show that ERT is slowly changing the prognosis. A long-term followup study of 17 patients with infantile-onset DGAA (up to 5.4 to 12 years of age) on ERT demonstrated improvements in cardiac measures of left ventricular mass index within five months of initiation of ERT [15]. This study also reported improvements in gross motor function, although residual muscle weakness with contractures, dysphagia with aspiration risk, hypernasal speech, and osteopenia were still present. In a study, 13 infants born in Taipei from 2010 through 2015 were diagnosed with infantile DGAA by a nationwide newborn screening program [16]. The mean age at the start of ERT was 12 days. Left ventricular function improved after three to four months of therapy. Institution of ERT has been reported as early as 18 hours after birth with resolved hypertrophic cardiomyopathy and normal neurodevelopment at 46 weeks [19]. Taken together, these data, along with our case, clearly show that cardiac hypertrophy associated with DGAA disease is a reversible phenomenon following the early onset of ERT. Further studies and the continuation of the followup of these patients are necessary to establish whether these results are stable over time and to evaluate further problems these children will encounter.

## 4. Conclusions

DGAA is an inherited storage disorder burdened by a very poor prognosis in untreated infantile-onset children. It is characterized by cardiomyopathy and severe generalized hypotonia, and the median age at death is nearly 8 months without treatment. The advent of ERT has improved clinical outcomes and survival. Its impact on cardiac function is still under study, but different reports in the literature are showing encouraging data. Hypertrophy of the myocardium, already found in the neonatal period, seems to be reversible with the onset of ERT, although it is currently impossible to establish whether these effects are long-lasting or not. However, early recognition of these conditions and prompt initiation of ERT is crucial to prevent progression of the disease and improve the outcomes.

## Data Availability

All data generated or analyzed during this study are included in this article and its supplementary information files. The data supporting this manuscript are from previously reported studies and datasets, which have been cited. The processed data are available in the supplementary file and from the corresponding author upon request.

## Abbreviations

CK: Creatine phosphokinase
CRIM: Cross-reactive immunological materials
DGAA: Acid alpha-glucosidase deficit
EKG: Electrocardiogram
GAA: Acid alpha-glucosidase
GSD: Glycogen storage disease
IVIG: Intravenous immunoglobulin
PD: Pompe disease.
